# The Duration of Proton Pump Inhibitor Therapy and the Risk of Small Intestinal Bacterial Overgrowth: A Systematic Review and Meta-Analysis

**DOI:** 10.3390/jcm14134702

**Published:** 2025-07-03

**Authors:** Alsu R. Khurmatullina, Dmitrii N. Andreev, Yury A. Kucheryavyy, Filipp S. Sokolov, Petr A. Beliy, Andrey V. Zaborovskiy, Igor V. Maev

**Affiliations:** 1 Department of Internal Disease Propaedeutics and Gastroenterology, Russian University of Medicine, 127473 Moscow, Russia; 2 Ilyinskaya Hospital, 143421 Krasnogorsk, Russia; 3 Department of Pharmacology, Russian University of Medicine, 127473 Moscow, Russia

**Keywords:** small intestinal bacterial overgrowth, proton pump inhibitors, PPI duration, meta-analysis, systematic review

## Abstract

**Background/Objectives:** Small intestinal bacterial overgrowth (SIBO) is frequently observed in patients treated with proton pump inhibitors (PPIs), yet the role of treatment duration in modulating SIBO risk remains unclear. This meta-analysis aims to evaluate the temporal association between PPI use duration and SIBO risk. **Methods:** Following the PRISMA 2020 guidelines, a systematic search was conducted across MEDLINE/PubMed, EMBASE, the Cochrane Library, the Russian Science Citation Index, and Google Scholar from 1985 to June 2025 and was previously registered in PROSPERO under the registration number CRD420251031719. Eligible studies included observational designs or clinical trials assessing SIBO in adult PPI users. **Results:** Twenty-nine studies (*n* = 3682 PPI patients; n = 2907 controls) were included. The pooled SIBO prevalence among PPI-treated patients was 36.839% (95% CI: 29.703–44.277), significantly higher than that among controls (19.887%; 95% CI: 12.027–29.399). PPI use was associated with increased SIBO risk (OR = 2.143; 95% CI: 1.446–3.175), with high heterogeneity (I^2^ = 77.61%). A duration-dependent trend was observed: our meta-regression analysis demonstrated a significant positive association between PPI treatment duration and SIBO prevalence with the regression coefficient 4.265% (95% CI: 1.827–6.384; *p* = 0.0024), indicating that each additional month of PPI therapy was associated with a 4.265 percentage increase in SIBO risk. **Conclusions:** PPI use significantly increases the risk of SIBO, with longer treatment durations associated with higher odds.

## 1. Introduction

Small intestinal bacterial overgrowth (SIBO) is a condition characterized by a significant increase in the number of bacteria in the small intestine, manifesting with gastrointestinal symptoms such as abdominal bloating, flatulence, nausea, abdominal pain, and diarrhea [1,2]. The global prevalence of SIBO varies widely, reported between 2.5% and 22%, with higher rates observed in older adults and individuals with comorbid conditions [3]. In individuals with gastrointestinal symptoms, the prevalence of SIBO can reach approximately 34% [4]. The clinical significance of SIBO lies in its ability to mimic the symptoms of several other GI diseases, complicating differential diagnosis in routine practice and often contributing to treatment resistance in associated conditions [5].

The long-term use of proton pump inhibitors (PPIs) is one of the most well-known risk factors for SIBO [2,6]. Changes in gastric pH affect the survival patterns of specific bacterial genera and species and promote their active migration throughout the GI tract [7]. This alteration in the microbial environment may reduce colonization resistance and facilitate overgrowth in the small intestine, particularly by oral-origin or facultative anaerobic bacteria [7]. The early meta-analysis (2018), which summarized 19 studies (n = 7055), demonstrated an increased risk of developing SIBO in patients taking PPIs (OR 1.71; 95% CI: 1.20–2.43) [8]. A large multicenter study (n = 1851) showed that symptoms characteristic of SIBO (diarrhea, bloating, abdominal pain, and discomfort) first appeared after a course of PPI therapy in 44.1% of cases [9]. Despite the well-documented association between PPI use and the development of SIBO, the relationship between the duration of PPI therapy and the risk of SIBO persistence remains insufficiently explored. While existing meta-analyses have established PPI use as a significant risk factor for SIBO [6,8], the impact of short-term versus long-term PPI exposure on bacterial overgrowth dynamics is less clear.

This meta-analysis aims to systematically evaluate the temporal relationship between PPI intake duration and SIBO risk, focusing on whether longer PPI exposure correlates with a higher incidence of SIBO.

## 2. Materials and Methods

### 2.1. Study Sources and Search

This systematic review adhered to the PRISMA 2020 guidelines [10] and was pre-registered in the PROSPERO registry (CRD420251031719) to ensure methodological transparency and minimize bias. A completed PRISMA-P checklist can be accessed from the Supplementary Materials, Table S1. The search was conducted across several electronic databases, including MEDLINE/PubMed, EMBASE, the Cochrane Library, the Russian Science Citation Index, and Google Scholar. Publications from 1 January 1985 to 1 June 2025 were considered. A comprehensive search strategy was developed using combinations of keywords and Boolean operators tailored to each database. The search strategy in MEDLINE/PubMed included the following terms: (“proton pump inhibitor”[tiab] OR “proton pump inhibitors”[tiab] OR PPI [tiab] OR omeprazole[tiab] OR pantoprazole[tiab] OR esomeprazole[tiab] OR lansoprazole[tiab]) AND (“small intestinal bacterial overgrowth”[tiab] OR “small bowel bacterial overgrowth”[tiab] OR SIBO[tiab] OR “bacterial overgrowth”[tiab]).

### 2.2. Study Selection

The study screening was carried out using Rayyan software. Two reviewers (A.R.K. and D.N.A.) independently evaluated titles and abstracts. Full-text screening followed for studies that appeared potentially relevant. Studies were deemed eligible if they met the following inclusion criteria: observational studies (cohort, case–control, cross-sectional) and clinical trials that evaluate the association between PPI use and SIBO risk in adults (≥18 years) with SIBO confirmed by hydrogen/methane breath testing or duodenal/jejunal culture. Studies had to provide sufficient data for effect size calculation. We excluded case reports, animal studies, pediatric populations, and studies involving patients with confounding conditions (e.g., prior GI surgery, Crohn’s disease, immunosuppression, tumor, systemic sclerosis). Duplicate datasets were thoroughly analyzed and only one study was chosen for the final analysis.

The risk of bias was assessed using the Newcastle–Ottawa scale for observational studies. Inter-observer agreement was quantified using Cohen’s kappa statistic, with the widely accepted interpretation scale: poor (κ ≤ 0.20), fair (κ = 0.21–0.40), moderate (κ = 0.41–0.60), good (κ = 0.61–0.80), and excellent agreement (κ > 0.80). The analysis encompassed both global inter-rater consistency and item-level agreement using these established thresholds.

### 2.3. Data Extraction

Two investigators (A.R.K. and D.N.A.) independently extracted data using standardized forms, documenting study characteristics such as publication year and geographic origin and detailed methodological information about SIBO diagnostic approaches. For each study, we recorded demographic parameters: the mean age of participants and cohort composition—including the number of patients receiving PPI therapy, corresponding control group sizes, and SIBO prevalence rates in both populations. More importantly, we extracted treatment duration data. The screening process was conducted by two medical experts (F.S.S. and P.A.B.) who independently assessed all identified studies through the AI-assisted Rayyan platform (https://www.rayyan.ai, accessed on 3 May 2025)), employing a three-stage evaluation procedure: initial title/abstract review against inclusion criteria and comprehensive full-text analysis of potentially relevant studies, followed by independent data extraction performed by two additional researchers (Y.A.K. and I.V.M.). To maintain rigorous data quality standards, we established verification protocols involving direct correspondence with study authors to clarify methodological details and formal solicitations for supplementary sensitivity testing data when original publications contained incomplete information.

### 2.4. Statistical Analysis

The primary focus was on calculating odds ratios (ORs). Also we analyzed pooled SIBO ratings in PPI-treated patients with 95% confidence intervals and assessed study heterogeneity using Cochran’s Q test and I^2^ statistics. Significant heterogeneity was defined as I^2^ > 50% and *p* < 0.05. Statistical analyses were performed in MedCalc 23.1.5 (Ostend, Belgium) on Microsoft Windows 11 (Microsoft Corporation, Redmond, WA, USA). To identify potential sources of heterogeneity, we conducted subanalyses comparing study quality (assessed by NOS scores) and diagnostic methods. Of particular importance was the temporal patterns, which were further analyzed using meta-regression implemented in Python 3.9.21 (Amsterdam, The Netherlands).

To evaluate potential publication bias, we employed both the visual inspection of funnel plot asymmetry and formal statistical tests (Begg–Mazumdar and Egger’s tests).

## 3. Results

### 3.1. Search Results

Our comprehensive literature search across electronic databases yielded an initial pool of 1014 potentially eligible scientific publications. After the duplicate search process 617 studies were removed. During the title/abstract screening process, we systematically excluded 338 records for the following reasons: 282 articles were irrelevant to our research focus, 44 represented case reports, and there were 10 reviews and 2 meta-analyses without original data.

**Figure 1:**
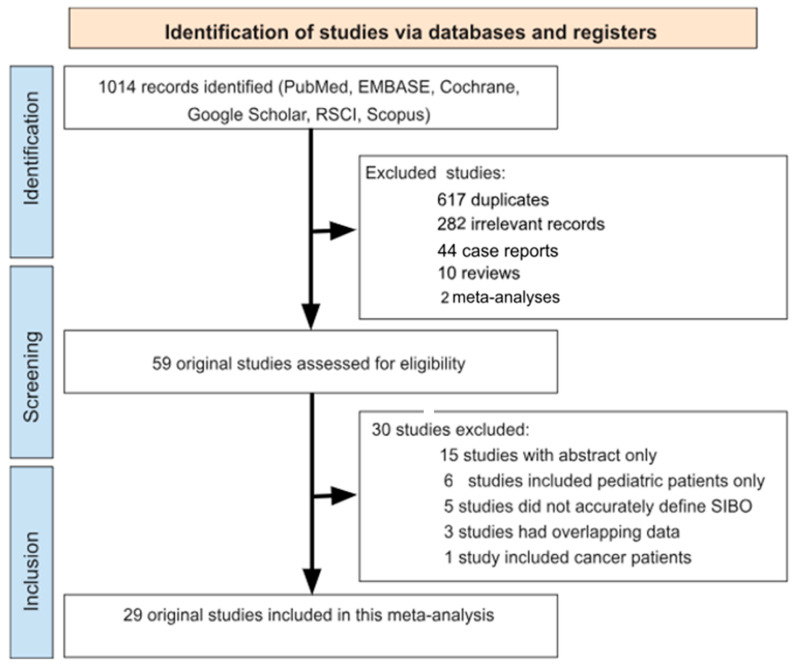
Flow chart detailing the study selection strategy.

The remaining 59 studies underwent meticulous full-text evaluation based on our inclusion criteria. This thorough assessment resulted in the exclusion of 30 additional articles that did not meet our methodological requirements, including 15 studies with abstracts only, 6 studies that included pediatric patients, 5 that did not accurately define SIBO cases, 3 studies that recruited a study population overlapping with that of another study and 1 study included cancer patients. The systematic review process and the number of papers identified in each stage are shown in Figure 1.

### 3.2. Characteristics of Included Studies

Final analysis included 29 studies [11,12,13,14,15,16,17,18,19,20,21,22,23,24,25,26,27,28,29,30,31,32,33,34,35,36,37,38,39]: 7 studies were conducted in the USA [17,19,22,23,26,28,35], 3 in the UK [12,15,16], 3 in Italy [18,20,36], 2 in Russia [24,39], 2 in Switzerland [11,14], 1 in Brazil [21], 1 in Argentina [25], 1 in Japan [27], 1 in Austria [29], 1 in Greece [30], 1 in India [31], 1 in Poland [32], 1 in China [33], 1 in Malaysia [34], 1 in Mexico [37], 1 in South Korea [38], and one in South Africa [13]. The total number of included PPI patients was 3682 and the total number of control patients was 2907. The gold-standard method for diagnostic aspirate culture was used in eight studies [11,13,14,16,19,23,26,30], the lactulose hydrogen breath test (LHBT) was used in eight studies [12,17,21,25,27,31,33,36,39], the glucose hydrogen breath test (GHBT) was used in seven studies [15,18,20,22,29,32,34,37,38], one study did not specify the substrate [24], the xylose breath test (XBT) was used in one study [28], and one study used 16S rRNA sequencing test [35]. This data is presented in Table 1.

### 3.3. Study Quality Evaluation and Reliability Analysis

Nine studies were of high methodological rigor (NOS 8–9), while twenty exhibited moderate limitations (NOS 6–7). Several key limitations affected study quality in our analysis. Most notably, 24.14% of included studies (7 out of 29) demonstrated restricted population representativeness due to small sample sizes (fewer than 30 PPI-treated patients). To ensure methodological rigor, we implemented robust quality control measures, quantifying inter-rater reliability through Cohen’s kappa coefficient. The results confirmed a strong consensus among reviewers, with κ = 0.65 (indicating substantial agreement) for study inclusion decisions and κ = 0.96 (reflecting near-perfect agreement) for data extraction processes.

### 3.4. Prevalence of SIBO in PPI Patients

**Figure 2:**
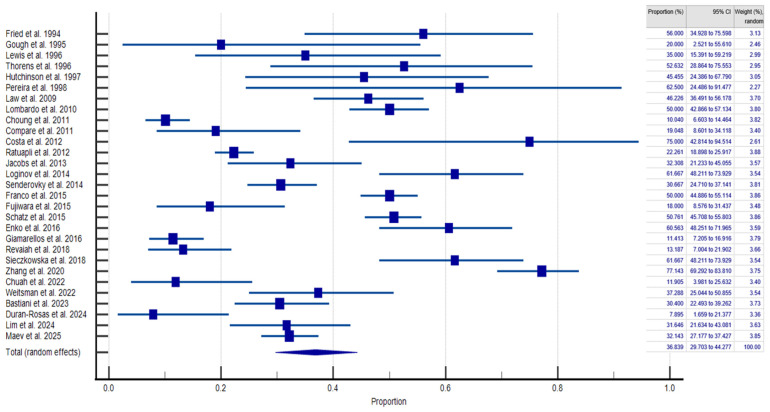
Forest plot showing the generalized prevalence of SIBO in PPI-treated patients [11,12,13,14,15,16,17,18,19,20,21,22,23,24,25,26,27,28,29,30,31,32,33,34,35,36,37,38,39].

**Figure 3:**
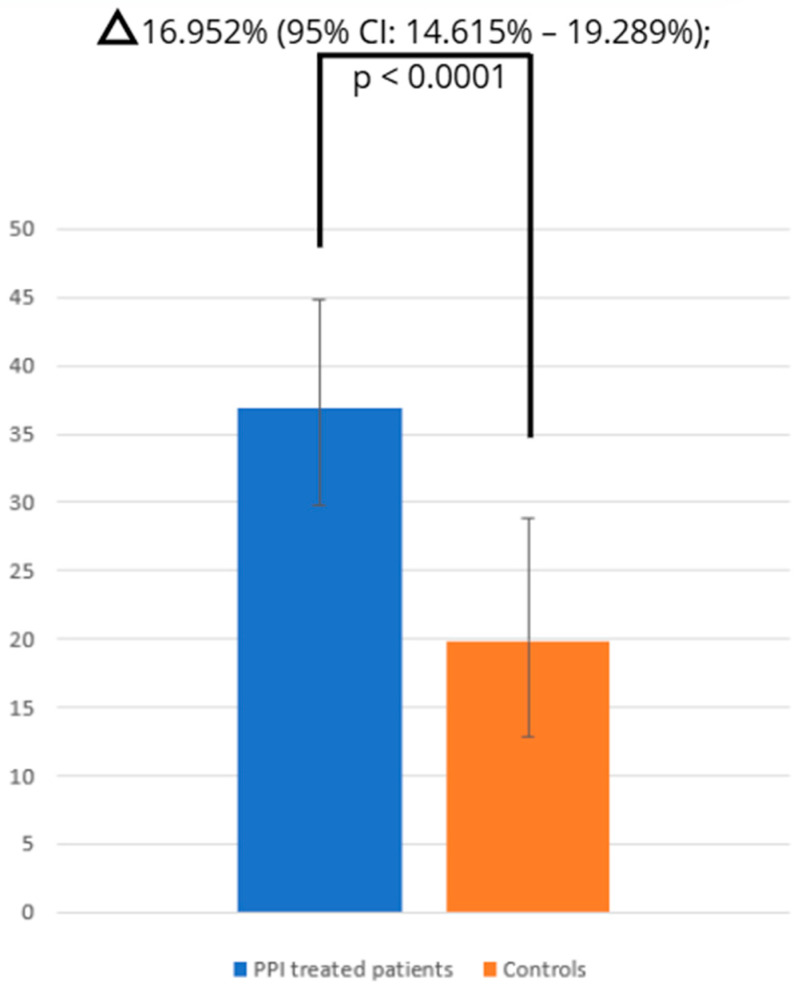
Comparison of SIBO prevalence between PPI and control groups.

**Figure 4:**
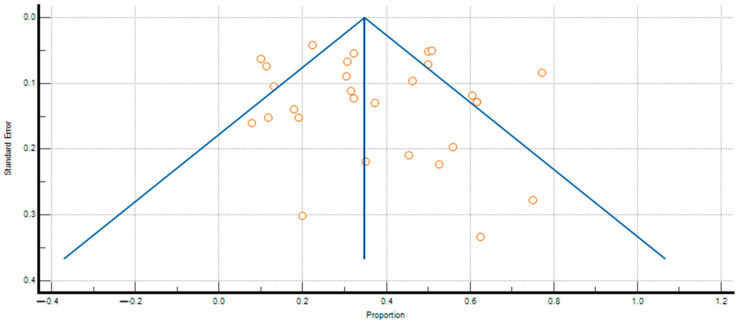
Funnel plot analysis of publication bias in SIBO prevalence among PPI-treated patients. Each point on the funnel plot represents an individual study, plotting its effect size against its precision.

The pooled prevalence of SIBO in PPI-treated patients and controls was 36.839% (95% CI: 29.703–44.277; Figure 2) and 19.887% (95% CI: 12.027–29.399; Figure 3), respectively, and heterogeneity was observed in both cases ($I_{PPI\;patients}^{2}$ = 94.93%; $I_{controls}^{2}$ = 96.66%; *p* < 0.0001). We assessed publication bias using funnel plot visualization and statistical tests. The funnel plot shows symmetrical study distribution, suggesting no significant bias (Figure 4). Both the Begg–Mazumdar test (*p* > 0.05) and Egger’s test (*p* > 0.05) confirmed this finding, demonstrating no evidence of substantial publication bias in our analysis. When analyzing studies that implied the gold-standard method (aspirate culture method), this tendency was also observed (35.877% (95% CI: 19.503–54.158) in PPI-treated patients and 10.783% (95% CI: 6.311–16.273) in controls).

The random-effects model revealed a significantly higher risk of SIBO among PPI-treated patients compared to controls (OR = 2.143; 95% CI: 1.446–3.175), with high heterogeneity (I^2^ = 77.61%; 95% CI: 65.03–85.67%; *p* < 0.0001).

### 3.5. Subanalyses

To evaluate the potential influence of PPI treatment duration on SIBO risk, we stratified the analysis into three distinct temporal categories: short-term use (less than one month—1), intermediate-term use (two to six months—2), and long-term use (more than six months—3). The analysis revealed a clear duration-dependent relationship, with progressively increasing odds ratios observed across the treatment periods. Patients receiving PPI therapy for less than one month demonstrated an odds ratio of 1.698 (95% CI: 0.185–15.626). The risk estimate rose to 4.002 (95% CI: 1.286–12.448) for those treated for one to six months. Most notably, long-term PPI use exceeding six months was associated with the highest risk elevation, yielding an odds ratio of 4.225 (95% CI: 1.432–12.464). These findings suggest a potentially significant temporal component in the development of PPI-associated SIBO, with longer treatment durations corresponding to progressively greater risk magnitudes.

To eradicate potential methodological influences on our findings, we performed additional stratified analyses according to diagnostic technique (Table 2). Among studies utilizing the gold-standard aspirate culture method, the prevalence of SIBO in PPI-treated patients was 35.877% (95% CI: 19.503–54.158). For investigations employing LHBT, the detected prevalence was slightly higher at 39.634% (95% CI: 27.027–52.984). Conversely, studies using GHBT demonstrated a more moderate prevalence estimate of 33.541% (95% CI: 21.561–46.711). This diagnostic-method-specific analysis reveals remarkable consistency across different assessment approaches, with all three principal diagnostic modalities yielding prevalence estimates within a relatively narrow range of approximately 33.5% to 39.6% (*p* < 0.0003 when comparing three methodologies between each other).

### 3.6. Meta-Regression

**Figure 5:**
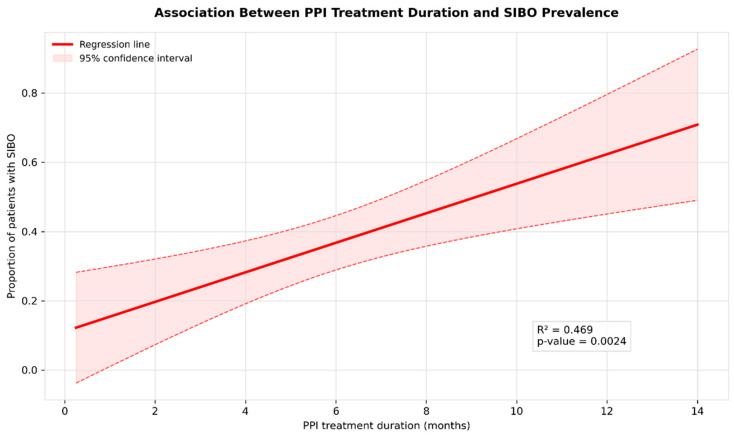
Meta-regression of SIBO prevalence by PPI treatment duration.

Our meta-regression analysis (Figure 5) demonstrated a significant positive association between PPI treatment duration and SIBO prevalence with the regression coefficient 4.265% (95% CI: 1.827–6.384; *p* = 0.0024), indicating that each additional month of PPI therapy was associated with a 4.265 percentage increase in SIBO risk.

### 3.7. Assessment of Publication Bias

To investigate the substantial heterogeneity observed in our primary analysis, we performed two subgroup analyses based on study quality as assessed by the NOS. The first subgroup included studies with moderate quality scores (NOS 6–7), which demonstrated a SIBO prevalence of 38.417% (95% CI: 28.714–48.609) among PPI users. The second subgroup comprised higher-quality studies (NOS 8–9), showing a slightly lower prevalence of 33.403% (95% CI: 23.192–44.470).

Notably, the difference between these two subgroups was relatively modest, with only a 5.014 percentage point (*p* = 0.0512) variation in SIBO prevalence estimates. More importantly, both subgroup estimates closely aligned with the overall pooled prevalence obtained in our overall analysis, reinforcing the robustness and consistency of our main findings. This concordance across different methodological quality tiers suggests that study quality, while contributing to some degree of heterogeneity, did not substantially alter the fundamental association between PPI use and SIBO risk.

## 4. Discussion

Our meta-analysis, encompassing 29 studies with a total of 3682 proton pump inhibitor (PPI) users and 2907 controls, reveals a significantly higher prevalence of small intestinal bacterial overgrowth (SIBO) among PPI-treated patients (36.8%; 95% CI: 29.7–44.3%) compared to controls (19.9%; 95% CI: 12.0–29.4%). Notably, a duration-dependent relationship was observed: patients undergoing PPI therapy for over six months exhibited the highest risk (OR = 4.225; 95% CI: 1.432–12.464). These findings underscore a robust association between prolonged PPI use and increased SIBO risk.

Several meta-analyses have investigated the association between PPI use and SIBO. Notably, our updated meta-analysis builds upon and significantly extends the findings of earlier studies conducted in 2012 [6] and 2016 [8]. While the previous analyses provided evidence of an increased risk of SIBO among PPI users, they were limited in scope and data range. Our study, incorporating data up to March 2025, offers a more comprehensive perspective on the relationship.

The 2012 meta-analysis included 11 studies and a total of 3134 patients. It reported an overall OR of 2.282 (95% CI: 1.238–4.205), suggesting a significant association between PPI use and SIBO. The 2016 analysis was more expansive, including 19 studies and 7055 patients. It found a slightly lower pooled OR of 1.71 (95% CI: 1.20–2.43). Nevertheless, neither prior study assessed the impact of PPI therapy duration.

In contrast, our 2025 meta-analysis includes 29 studies published between 1985 and 2025, totaling 9496 subjects. We utilized a broader set of databases—MEDLINE, EMBASE, Cochrane, Google Scholar, and the Russian Science Citation Index. All included studies were assessed using the Newcastle–Ottawa Scale, with inter-rater agreement measured using the κ-statistic. Our findings indicate a pooled OR of 2.143 (95% CI: 1.446–3.175), reinforcing the association between PPI use and an increased risk of SIBO. Unlike the previous meta-analyses, our subgroup analysis did not reveal significant variation in effect size across diagnostic methods, suggesting a more consistent risk regardless of how SIBO is identified. Additionally, we conducted meta-regression analyses, which revealed that the duration of PPI therapy is a significant predictor of SIBO risk—longer treatment periods were associated with higher odds of developing SIBO, a novel finding not previously reported.

The clinical implications are significant, given that SIBO is associated with various conditions, including irritable bowel syndrome, inflammatory bowel disease, chronic pancreatitis, liver cirrhosis, and metabolic disorders like diabetes and non-alcoholic fatty liver disease [40,41,42,43,44]. The widespread prescription of PPIs for acid-related disorders—such as gastroesophageal reflux disease (GERD) and peptic ulcers—further amplifies the relevance of our findings [45]. PPIs are often recommended as the first-line treatment for these conditions, with therapy durations ranging from 4 to 8 weeks [46,47]. However, in cases of chronic or recurrent GERD, maintenance therapy may extend up to six months or longer [46], and for Barrett’s esophagus, lifelong PPI therapy is commonly advised [48]. All of these patients have a high risk of SIBO as a consequence of intestinal symptoms, which was shown in recent works [7,49].

International real-world data indicates that prolonged PPI use beyond two months is common. Use varies regionally—for example, in Australia 25% of initial PPI users are on maintenance regimens [50], while among older adults in Portugal nearly 79% exceed the guideline-recommended 8 weeks [51]. Over-the-counter availability further complicates exposure assessment, contributing between 1 and 10% of national PPI consumption in some countries [52]. These patterns underscore that a substantial proportion of patients fall into our intermediate- and long-term exposure subgroups.

Given the potential for long-term PPI use to increase SIBO risk, it is imperative to implement deprescribing protocols where appropriate. While such protocols have been developed, their integration into clinical practice remains limited in many healthcare systems [53,54,55]. Enhancing awareness and application of these strategies could mitigate the risk of SIBO and its associated complications in patients requiring prolonged PPI therapy.

Our meta-analysis has several limitations. First, the lack of standardized diagnostic criteria for SIBO likely contributes to heterogeneity, as studies employed varying thresholds for breath tests and differing techniques for aspirate cultures. Second, the inclusion of studies from 15 countries introduces potential geographical variations in microbiome composition, dietary habits, and PPI prescribing practices. Third, a notable proportion (24.14%) of the included studies had small sample sizes (<30 PPI-treated patients), which may limit the precision of subgroup analyses. Fourth, while nine studies were of high methodological quality (NOS 8–9), the majority (n = 20) had moderate limitations, particularly in patient selection and comparability. Finally, dietary influences may have impacted the observed associations.

Despite these limitations, our study represents the most extensive and methodologically rigorous meta-analysis to date on PPI-associated SIBO. These findings underscore the importance of clinical attention to long-term PPI users and suggest potential strategies for risk management. Future research should prioritize prospective cohort studies with standardized diagnostic protocols and severe adjustment for potential variables to further clarify this relationship and explore therapeutic interventions.

## 5. Conclusions

Our meta-analysis demonstrates that SIBO is a frequent complication in PPI-treated patients, affecting approximately 36.8% of them. Given the significant gastrointestinal and systemic consequences associated with SIBO, clinicians should maintain strong suspicion of this condition in patients receiving prolonged PPI treatment.

## Figures and Tables

**Table 1:** Characteristics of selected studies.

Study, Year	Country	Study Design	SIBO Diagnostic Method	Mean Age	PPI-Treated Patients	Period of PPI Treatment	Control Patients	NOS
Fried et al., 1994 [11]	Switzerland	Case–control	Aspirate culture	50.6	25	2	15	6
Gough et al., 1995 [12]	UK	Cohort	LHBT	55	10	1	n/d	7
Lewis et al., 1996 [13]	South Africa	Cohort	Aspirate culture	43.1	20	1	n/d	6
Thorens et al., 1996 [14]	Switzerland	Case–control	Aspirate culture	42	19	1	18	6
Hutchinson et al., 1997 [15]	UK	Case–control	GHBT	78.6	22	1	22	7
Pereira et al., 1998 [16]	UK	Case–control	Aspirate culture	76	8	2	6	7
Law et al., 2009 [17]	USA	Case–control	LHBT	43.7	106	n/d	449	8
Lombardo et al., 2010 [18]	Italy	Case–control	GHBT	37.7	200	3	50	6
Choung et al., 2011 [19]	USA	Case–control	Aspirate culture	53	249	n/d	421	6
Compare et al., 2011 [20]	Italy	Cohort	GHBT	36	42	3	n/d	7
Costa et al., 2012 [21]	Brazil	Case–control	LHBT	38.8 ± 12.62	12	n/d	11	8
Ratuapli et al., 2012 [22]	USA	Case–control	GHBT	60.9	566	n/d	625	7
Jacobs et al., 2013 [23]	USA	Case–control	Aspirate culture	43	65	n/d	85	8
Loginov et al., 2014 [24]	Russia	Cohort	HBT	58 ± 2.1	60	3	n/d	7
Senderovky et al., 2014 [25]	Argentina	Cohort	LHBT	57.5	225	n/d	n/d	7
Franco et al., 2015 [26]	USA	Cohort	Aspirate culture	52	384	3	n/d	8
Fujiwara et al., 2015 [27]	Japan	Cohort	LHBT	n/d	50	n/d	n/d	6
Schatz et al., 2015 [28]	USA	Cohort	XBT	57.4	394	n/d	n/d	6
Enko et al., 2016 [29]	Austria	Case–control	GHBT	44	71	3	38	7
Giamarellos et al., 2016 [30]	Greece	Case–control	Aspirate culture	64.4	184	2	713	8
Revaiah et al., 2018 [31]	India	Case–control	LHBT	41.71 ± 13.17	91	2	56	6
Sieczkowska et al., 2018 [32]	Poland	Case–control	GHBT	n/d	60	3	62	6
Zhang et al., 2020 [33]	China	Case–control	LHBT	n/d	140	n/d	60	7
Chuah et al., 2022 [34]	Malaysia	Case–control	GHBT	48 ± 16 years	42	2	58	9
Weitsman et al., 2022 [35]	USA	Case–control	16S rRNA Sequencing	61.5 ± 13.6	59	n/d	118	7
Bastiani et al., 2023 [36]	Italy	Case–control	LHBT	n/d	125	3	100	8
Duran-Rosas et al., 2024 [37]	Mexico	Cohort	GHBT	25.18 ± 6.5	38	1	n/d	7
Lim et al., 2024 [38]	South Korea	Cohort	GHBT	71.83 ± 8.80	79	2	n/d	8
Maev et al., 2025 [39]	Russia	Cohort	LHBT	38.7 ± 8.9	336	2	n/d	8

**Table 2:** Subgroup analysis of SIBO risk and prevalence among PPI users stratified by diagnostic method and study quality.

Subgroup Analysis	Pooled OR (95% CI)	Heterogeneity I^2^ (%)	Pooled Prevalence (95% CI)	Heterogeneity I^2^ (%)	*p*
SIBO diagnostic method					
Aspirate culture	1.727 (1.304–2.287)	46.87	35.877% (19.503–54.158)	95.72	0.0025
GHBT	2.508 (0.995–6.323)	87.42	33.541% (21.561–46.711).	93.78	0.0451
LHBT	1.939 (0.811–4.634)	81.51	39.634% (27.027–52.984)	94.88	0.0055
Overall	2.143 (1.446–3.175)	77.61	36.839% (29.703–44.277)	94.93	<0.0001
Study quality (NOS scores)					
6–7	2.962 (1.717–5.112)	79.12	38.417% (28.714–48.609)	95.56	<0.0001
8–9	1.211 (0.736–1.992)	64.11	33.403% (23.192–44.470)	93.58	<0.0001

## Data Availability

No new data were created or analyzed in this study. Data sharing is not applicable to this article.

## Supplementary Materials

The following supporting information can be downloaded at https://www.mdpi.com/article/10.3390/jcm14134702/s1. Table S1. PRISMA NMA Checklist of Items to Include When Reporting a Systematic Review Involving a Network Meta-analysis.
